# Dysfunction of Cellular Proteostasis in Parkinson’s Disease

**DOI:** 10.3389/fnins.2019.00457

**Published:** 2019-05-10

**Authors:** Šárka Lehtonen, Tuuli-Maria Sonninen, Sara Wojciechowski, Gundars Goldsteins, Jari Koistinaho

**Affiliations:** ^1^A.I. Virtanen Institute for Molecular Sciences, University of Eastern Finland, Kuopio, Finland; ^2^Neuroscience Center, Helsinki Institute of Life Science, University of Helsinki, Helsinki, Finland

**Keywords:** proteostasis, alpha-synuclein, refolding, ER stress, UPR, protein disulfide isomerase

## Abstract

Despite decades of research, current therapeutic interventions for Parkinson’s disease (PD) are insufficient as they fail to modify disease progression by ameliorating the underlying pathology. Cellular proteostasis (protein homeostasis) is an essential factor in maintaining a persistent environment for neuronal activity. Proteostasis is ensured by mechanisms including regulation of protein translation, chaperone-assisted protein folding and protein degradation pathways. It is generally accepted that deficits in proteostasis are linked to various neurodegenerative diseases including PD. While the proteasome fails to degrade large protein aggregates, particularly alpha-synuclein (α-SYN) in PD, drug-induced activation of autophagy can efficiently remove aggregates and prevent degeneration of dopaminergic (DA) neurons. Therefore, maintenance of these mechanisms is essential to preserve all cellular functions relying on a correctly folded proteome. The correlations between endoplasmic reticulum (ER) stress and the unfolded protein response (UPR) that aims to restore proteostasis within the secretory pathway are well-established. However, while mild insults increase the activity of chaperones, prolonged cell stress, or insufficient adaptive response causes cell death. Modulating the activity of molecular chaperones, such as protein disulfide isomerase which assists refolding and contributes to the removal of unfolded proteins, and their associated pathways may offer a new approach for disease-modifying treatment. Here, we summarize some of the key concepts and emerging ideas on the relation of protein aggregation and imbalanced proteostasis with an emphasis on PD as our area of main expertise. Furthermore, we discuss recent insights into the strategies for reducing the toxic effects of protein unfolding in PD by targeting the ER UPR pathway.

## Introduction

In Parkinson’s disease (PD), the loss of dopaminergic (DA) neurons in the *substantia nigra pars compacta* (SNpc) and subsequent loss of dopamine in the striatum leads to typical motor impairments in PD, such as bradykinesia, rigidity, rest tremor, and postural instability. There are various non-motor symptoms also associated with PD including anosmia, gastrointestinal motility issues, sleep disturbances, sympathetic denervation, anxiety, and depression. These non-motor symptoms generally precede the motor impairments by years (Kalia and Lang, 2015). The presence of Lewy bodies (LBs) with an accumulation of the protein alpha-synuclein (α-SYN) is one of the pathological hallmarks in PD (Kalia and Lang, 2015; Sveinbjornsdottir, 2016). There is not yet a cure, although, treatments are available to relieve symptoms. Approximately 20 PD-associated genes have been identified to date even though most cases are late onset and sporadic with no evidence for inheritance or genetic cause (Klein and Westenberger, 2012). The phenotypes of both the sporadic and familial forms are essentially indistinguishable, implying that they might share common underlying mechanisms. Moreover, many similarities including protein misfolding and aggregation are also commonly seen in other neurodegenerative diseases. While the exact role of protein aggregation in disease pathology is still under debate, discovering these similarities offers hope for therapeutic advances that could affect many diseases simultaneously. In this review, we summarize recent progress in the studies on the mechanism of endoplasmic reticulum (ER) stress-induced unfolded protein response (UPR) in PD, how protein aggregation relates to imbalanced proteostasis and how to remedy the toxic effects of protein unfolding in PD by targeting the ER UPR pathway.

## Description of Cellular Proteostasis Deficits in PD

### Physiological Role of α-SYN and Aggregation

α-SYN is a small (14 kDa) protein that is highly expressed in neurons but can also be found in peripheral tissues and blood (Witt, 2013; Malek et al., 2014). A recent report also demonstrated its expression in astrocytes (di Domenico et al., 2019). The physiological function of α-SYN remains mostly undefined (Devine et al., 2011; Liu et al., 2012; Kalia and Kalia, 2015), nevertheless, the involvement in synaptic maintenance, mitochondrial homeostasis, dopamine metabolism, and chaperone activity has been studied. Typically, α-SYN is a monomer with three structural regions (Villar-Piqué et al., 2016). The N-terminal domain (1–60) contains a multi-repeated consensus sequence (KTKEGV) and is responsible for the membrane-binding capacity. The central domain (61–95) is known as the non-amyloid-beta component and contains a highly hydrophobic motif which is involved in α-SYN aggregation. The C-terminal domain’s (96–140) proline residues have been found to be acidic. The exact native structure of α-SYN is not completely established, but several studies have described it as a soluble protein with a disordered monomeric structure (Binolfi et al., 2012; Fauvet et al., 2012; Waudby et al., 2013). In addition, soluble tetramers have been identified (Bartels et al., 2011), but the physiologically relevant structure of α-SYN may differ depending on the cellular location and environment. The non-amyloid-beta domain of α-SYN is prone to aggregate, but in its native structure, it appears to be protected by the N- and C-termini (Bertoncini et al., 2005). Changes in environment, mutations and/or post-translational modifications (PTMs) may disrupt the native conformation of α-SYN and induce misfolding and aggregation.

Initially, α-SYN was identified in the nucleus, but this is still in dispute (Huang et al., 2011). It has been proposed that the nuclear protein TRIM28 regulates its translocation into the nucleus and α-SYN may play a role in transcription regulation and histone acetylation (Kontopoulos et al., 2006; Rousseaux et al., 2016). Several studies have shown that PD associated mutations, PTMs and oxidative stress can increase the nuclear localization of α-SYN (Kontopoulos et al., 2006; Xu et al., 2006; Schell et al., 2009; Gonçalves and Outeiro, 2013; Fares et al., 2014). In addition, animal and cellular models and patient studies have shown altered activation of transcription factors upon α-SYN translocation. These include decreased activation of the mitochondrial biogenesis factor PGC-1α, reduced activation of the autophagy-lysosomal pathway (ALP) transcription factor EB (TFEB), and increased activation of calcineurin and subsequent nuclear translocation of nuclear factor of activated T cells (Decressac et al., 2013; Ryan et al., 2013; Luo et al., 2014; Eschbach et al., 2015).

α-SYN is associated with several neurodegenerative disorders, collectively known as synucleinopathies (Wong and Krainc, 2017). α-SYN fibrils are the main component found in LB and Lewy neurites (LNs) in PD and dementia with Lewy bodies (DLBs). LBs are spherical aggregates of α-SYN found in neuronal cell bodies, while LNs are aggregate structures found in neuronal dendrites and axons. Structurally, LBs are made up of insoluble eosinophilic amyloid that is surrounded by fibrils of α-SYN which are typically ubiquitinated (Beyer et al., 2009). In sporadic PD, α-SYN accumulates in neuronal cell bodies and processes resulting in LBs and LNs, respectively. Duplication of *SNCA* results in late-onset autosomal dominant forms of PD and triplication results in early-onset PD (Singleton et al., 2003). This demonstrates that α-SYN levels correlate with the onset of PD. In addition, other mutations causing familial PD, like mutations in leucine-rich repeat kinase 2 (LRRK2), can develop LB pathology (Zimprich et al., 2004). When comparing the pathology of DLB, there are some similarities with PD, but the clinical symptoms are closer to Alzheimer’s disease (Spillantini et al., 1998). In PD, the substantia nigra (SN) is affected, while in DLB the pathology is seen in the cortex. In addition to PD and DLB, α-SYN accumulation is present in multiple system atrophy (MSA) and pure autonomic failure (PAF). In MSA the α-SYN inclusions are present in the cytosol of oligodendrocytes. Mutations in α-SYN can cause both PD and MSA symptoms (Fanciulli and Wenning, 2015). LBs and LNs are found in the sympathetic nervous system in PAF (Arai et al., 2000). In addition to synucleinopathies, α-SYN toxicity has been associated with lysosomal storage disorders such as Gaucher’s disease, a rare genetic disorder characterized by the deposition of glucocerebroside in cells of the macrophage-monocyte system (Blanz and Saftig, 2016). Mutations in *GBA1*, which encodes glucocerebrosidase (GCase) and causes Gaucher’s disease, are the most common risk factors for PD. Some patients carrying these mutations may develop parkinsonism, a clinical syndrome characterized by movement disorders commonly seen in PD, with LB pathology.

Oligomers and fibrils are considered to be the toxic species of α-SYN, but there remains some disagreement regarding their toxicity. Several studies have suggested that soluble oligomers are more toxic than fibrils or aggregates. For example, increased levels of soluble oligomers have been identified in α-SYN transgenic mice and in PD and DLB patient brains. Oligomeric α-SYN caused more severe DA neuron loss than fibrils in rats (Sharon et al., 2003; Winner et al., 2011). By contrast, some studies have shown fibrils to be more toxic compared to the oligomers and caused increased motor impairment, DA cell loss and synaptic impairment (Peelaerts et al., 2015).

In neurons, α-SYN is known to localize in presynaptic terminals and regulate synaptic transmission. The release of neurotransmitters requires cycles of soluble *N*-ethylmaleimide-sensitive factor attachment protein receptor (SNARE)-complex assembly and disassembly. α-SYN has been shown to bind to SNARE protein synaptobrevin-2/vesicle-associated membrane protein 2 (VAMP2) and promote SNARE-complex assembly (Burré et al., 2010). The same study also demonstrated that triple knockout mice developed neurological impairments and had decreased SNARE assembly. Subsequently, it was described that α-SYN promotes vesicle-clustering activity, which is dependent on the interaction of α-SYN with synaptobrevin-2/VAMP2 and anionic lipids (Diao et al., 2013). These studies suggest that the major cellular function of α-SYN are interactions of α-SYN with cell membranes, and that the cytosolic state may be transient.

While α-SYN is normally localized in presynaptic terminals, the oligomers and aggregates can be found in cell bodies and neurites, as well as in other cell types, including astrocytes which indicates a widespread toxic action. The pathological effects of α-SYN can affect the function of several different organelles, including synaptic vesicles, mitochondria, lipid bilayers, cell’s cytoskeleton, ER, Golgi, proteasomes, lysosomes, and nucleus. α-SYN oligomers can disrupt the SNARE complex formation, dopamine release and synaptic-vesicle motility (Choi et al., 2013; Wang et al., 2014). Increased levels of α-SYN can also decrease the synaptic-vesicle recycling-pool size and mobility leading to a disrupted neurotransmitter release (Nemani et al., 2010; Scott and Roy, 2012). It was discovered that dopamine neurotransmission can be disrupted by high levels of α-SYN. Transgenic mice overexpressing α-SYN showed a DA terminal loss, deficient release and altered synaptic-vesicle distribution (Masliah et al., 2000; Janezic et al., 2013). Moreover, the reduction in dopamine reuptake and defective dopamine transporter function has been linked to increased levels of α-SYN (Lundblad et al., 2012).

The homeostasis of mitochondria can be disrupted by α-SYN toxicity. Mice with an A53T α-SYN mutation have increased mitochondrial DNA damage and upregulated mitophagy (Martin et al., 2006; Choubey et al., 2011; Chen et al., 2015). In contrast, a recent study showed delayed mitophagy in PD patient neurons caused by abnormal accumulation of Miro protein (Shaltouki et al., 2018). α-SYN oligomers also reduced axonal mitochondria transport in induced pluripotent stem cell (iPSC)- derived neurons (Prots et al., 2018). Recent studies have also shown that α-SYN translocated to the mitochondrial matrix and caused impairment of complex I leading to decreased ATP synthesis and increased reactive oxygen species (ROS) production (Martínez et al., 2018). These results suggest that α-SYN can disrupt the mitochondrial homeostasis in several ways.

α-SYN oligomers can interact with and permeabilize lipid membranes causing structural alterations of the intracellular and plasma membranes, increase of intracellular calcium levels, and activation of calpain (van Rooijen et al., 2010; Melachroinou et al., 2013; Ronzitti et al., 2014). Additionally, α-SYN oligomers can inhibit tubulin polymerization and impair neurite network morphology and overexpression in cultured cells and cause microtubule destabilization and neurite degeneration (Lee et al., 2006; Chen et al., 2007; Prots et al., 2013). α-SYN fibrils have also been shown to impair axonal transport of autophagosomes and endosomes but the fibrils didn’t affect the transport of synaptophysin or mitochondria (Volpicelli-Daley et al., 2014). However, a recent study found that α-SYN oligomers disrupted anterograde axonal transport of mitochondria and caused subcellular changes in transport-regulating proteins in iPSC- derived neurons (Prots et al., 2018).

### Major Pathways of Alpha-Synuclein Clearence in PD

The protein degradation system is part of a protein quality control machinery which clears non-essential misfolded, or damaged proteins. The two major protein degradation systems are the ubiquitin-proteasome pathway (UPP) and ALP. Both are affected in synucleinopathies. These pathways have been shown to be responsible for degrading α-SYN, and failure in one or both can lead to accumulation. The progressive accumulation of α-SYN typical in PD can be linked to the disruption of the UPP by aggregation (Lindersson et al., 2004) as well as different types of autophagy (Winslow et al., 2010; Malkus and Ischiropoulos, 2012). It has been shown that aggregated α-SYN can bind to the membrane proteins of lysosomes and block their function (Malkus and Ischiropoulos, 2012) as well as inhibit certain enzymatic activity domains of proteasomes (Lindersson et al., 2004). α-SYN also inhibits the expression of proteins relevant to autophagosome assembly leading to inefficient removal of aggregated proteins due to impairment in macroautophagy (Winslow et al., 2010).

#### The Ubiquitin-Proteasome Pathway

**FIGURE 1:**
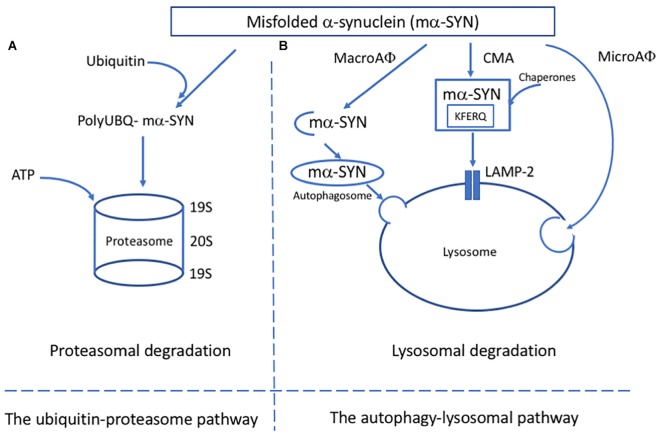
Major pathways of alpha-synuclein clearance in PD. **(A)** In the ubiquitin-proteosome pathway, mα-SYN is tagged with ubiquitin molecules and transferred to the proteosome complex for ATP dependant degradation. **(B)** In the autophagy-lysosomal pathway (ALP), three different pathways have been described: microautophagy (microAφ), macroautophagy (macroAφ) and chaperone-mediated autophagy (CMA). While evidence linking microAφ and mα-SYN is still missing, in macroAφ, mα-SYN is sequestered by a double membrane-organelles called autophagosomes before fusion with lysosome. In CMA, mα-SYN bind to protein chaperones, which help to target them directly to the lysosome for enzymatic degradation.

In the UPP, short-living proteins that are coupled with ubiquitin molecules are degraded by proteasomes (Pickart, 2001; Glickman and Ciechanover, 2002) (Figure 1A). The first evidence of UPP failure in PD came from post-mortem studies that used enzymatic assays to evaluate proteasome activity in brain tissues. These studies showed a significant decrease in the chymotrypsin-like and trypsin-like proteasome activity in the SN of PD patients in comparison to age-matched controls. No evidence of defective proteasome activity was seen in other brain regions but rather increased activity was observed in the unaffected areas (Furukawa et al., 2002). These studies suggested that reduced proteasome activity is specific for certain brain regions, like SN. In contrast, it is probable that the decreased activity of proteasomes could be a consequence of the neurodegeneration in this region.

Reduced levels of proteasome subunits have been observed in PD patients. Several genes that code for proteasome subunits were downregulated in the SN of PD patients and were linked to reduced levels of 20S proteasome core, α-subunit and 19S regulatory caps (Furukawa et al., 2002; McNaught et al., 2002a, 2003; Grünblatt et al., 2004; Chu et al., 2009; Bukhatwa et al., 2010).

Some studies have demonstrated altered proteasome function in peripheral blood cells of PD patients, but the results were significant only in patients treated with L-DOPA and dopamine agonists (Blandini et al., 2006; Ullrich et al., 2010). This indicates that dopamine levels can alter the proteasome function and is supported by animal and *in vitro* models (Yoshimoto et al., 2005; Berthet et al., 2012).

Studies with disease models have implicated the dysfunction of UPP in PD. Treatment with proteasome inhibitor lactacystin leads to dose-dependent neurodegeneration and formation of ubiquitin and α-SYN positive inclusions in α-SYN-eGFP transfected mouse primary neurons, rat ventral mesencephalic primary neurons, and cultured PC12 cells (McLean et al., 2001; Rideout et al., 2001; McNaught et al., 2002b). McNaught and colleagues used a systemic application of proteasome inhibitor in rats which led to motor deficits and main pathological features typical for PD (McNaught et al., 2004). Also, neurons were found to contain α-SYN and ubiquitin-positive inclusion bodies. Since then, this model has been challenged due to several laboratories inability to replicate the model (more information, see review, Bentea et al., 2017). Besides these studies, toxin-based animal and cellular models have implicated a link between sporadic PD and UPP failure. The toxin 1-methyl-4-phenyl-1,2,3,6-tetrahydropyridine (MPTP) appears to target specifically those neurons that are involved in PD. MPTP can easily cross the blood–brain barrier and then is metabolized by astrocytes to become 1-methyl-4-phenylpyridinium (MPP++) ion which is also toxic. MPP+ is released from astrocytes and taken up by DA neurons. MPTP administration causes nigral cell loss, striatal dopamine loss and behavioral deficits (Meredith and Rademacher, 2011). Several *in vitro* experiments have shown decreased proteasome activity after exposure of pesticides and environmental toxins linked to PD (Wang et al., 2006; Caneda-Ferrón et al., 2008; Chou et al., 2010). Consistent with these findings, *in vivo* studies showed reduced proteasome activity after rotenone and MPTP administration (Fornai et al., 2005; Betarbet et al., 2006). The UPP impairment caused by MPTP was alleviated in mice lacking α-SYN suggesting that it increases the detrimental effects of MPTP on the UPP (Fornai et al., 2005).

Numerous *in vitro* studies with purified proteins or cell culture systems, have demonstrated that mutant or wild-type α-SYN can inhibit 20S or 26S proteasome activity, especially in the case of oligomer or fibril formation. In PC12 cells expressing human mutant A53T α-SYN, cells exhibited accumulation of cytoplasmic ubiquitinated aggregates corresponding with decreased proteasomal chymotrypsin-like activity measured from cell lysates (Stefanis et al., 2001). The finding was confirmed by another study using the same cell line which showed that chymotrypsin-like, trypsin-like and caspase-like activities of the proteasome were all decreased (Tanaka et al., 2001). Also, mutant α-SYN increased cell death in the presence of a proteasome inhibitor. In M17 neuroblastoma cells, mutant α-SYN (A30P or A53T) increased sensitivity to proteasome inhibitors by decreasing proteasome function measured by a GFP reporter system (Petrucelli et al., 2002). These studies indicated reduced proteasome function related to mutant α-SYN, but not to wild-type. However, Snyder et al. (2003) showed inhibition of the proteasome with overexpressing wild-type α-SYN in neuroblastoma M17 cells. A study with yeasts revealed impairment of proteasome-mediated protein degradation in cells expressing wild-type and mutant (A30P) α-SYN (Chen et al., 2005). The cells expressing α-SYN also exhibited a decrease in chymotrypsin-like activity, but no other proteolytic activity of the proteasome was altered.

Contrary to these studies, overexpression of wild-type or mutant (A30P, A53T) α-SYN in PC12 cells or transgenic mice did not result in dysfunction of the UPP (Martìn-Clemente et al., 2004). In a more recent study, Zondler et al. (2017) demonstrated that the impairment of proteasome activity by α-SYN is dependent upon the cellular background. In this study, recombinant α-SYN oligomers and fibrils *in vitro* or transient expression of wild-type or mutant (A30P, A53T) α-SYN in U2OS ps 2042 [Ubi(G76V)-GFP] cells did not affect 20S proteasome function. In contrast, in DA SH-SY5Y and PC12 cells, stable expression of both wild-type and mutant α-SYN resulted in impairment of the chymotrypsin-like 20S/26S proteasomal protein cleavage.

The proposed mechanisms of how α-SYN inhibits the activity of the proteasome may be by direct binding to the S6′ or the Rpt5 subunit of the 19S proteasome, or to the β5 subunit of the 20S proteasome (Ghee et al., 2000; Snyder et al., 2003; Lindersson et al., 2004). In addition, different α-SYN species have been implicated in UPP dysfunction. PC12 cells expressing wild-type or mutant α-SYN produce soluble intermediate sized oligomers that associate with the 26S proteasome and increase in amount after treatment with proteasomal inhibitor, indicating specific degradation of the 26S proteasome (Emmanouilidou et al., 2010). In fact, the expression of α-SYN leads to inhibition of all proteasome activities. This study suggested that only a subset of soluble cell-derived α-SYN oligomers are targeted to the 26S proteasome for degradation. Simultaneously, these species can inhibit the proteasome function.

#### The Autophagy-Lysosomal Pathway

The ALP is responsible for degrading long-lived proteins, cellular components and organelles through the lysosomal compartment (Parzych and Klionsky, 2014). The ALP has two main purposes: to clear deleterious intracellular components and recycle macromolecules from organelles and proteins to guarantee proteome renewal. Depending on the delivery method, ALP can be divided into three pathways: macroautophagy, chaperone-mediated autophagy (CMA) and microautophagy (Figure 1B). Because of the lack of evidence linking microautophagy to α-SYN, the focus here is on macroautophagy and CMA.

Macroautophagy is an evolutionary and highly conserved process and the best known of the three autophagic mechanisms (Parzych and Klionsky, 2014; Bento et al., 2016). After the discovery of autophagy-related genes (Atg), the molecular pathway of macroautophagy has been well-characterized. Macroautophagy involves the formation, elongation, and nucleation of double-membrane organelles called autophagosomes that sequester the substrate before fusion with lysosomes. CMA is a particular system based on the recognition of a specific amino acid sequence (KFERQ) (Dice, 1990). The cytosolic chaperone heat shock cognate protein of 70 kDa (hsc70) recognizes the specific motif and translocates the substrate into the lysosome membrane where it interacts with the lysosome-associated membrane protein type 2A (LAMP2A) (Cuervo and Dice, 1996). The final step of the translocation requires the presence of lysosome-associated hsc70 (lys-hsc70) which disassembles LAMP2A into monomers and initiates a new cycle of substrate uptake and degradation (Agarraberes et al., 1997).

Several genetic factors related to PD are involved or interact with ALP (Gan-Or et al., 2015). Mutations in *GBA1* that encode the lysosomal hydrolase GCase can lead to lysosomal dysfunction and disruption of autophagy. For example, SCARB2, which encodes the lysosomal integral membrane protein type 2 and interacts with GCase, has been associated with a reduced risk for PD (Gan-Or et al., 2015). Mutations in the ATP13A2 (*PARK9*) gene, encoding a lysosomal ATPase, causes Kufor-Rakeb syndrome, a rare form of atypical, juvenile-onset autosomal recessive parkinsonism with pyramidal neurodegeneration and dementia.

Along with mutations in genes coding for lysosomal components, other PD-related mutations have been implicated in the process of autophagy. Mutations in the gene encoding vacuolar protein sorting-associated protein 35 (VPS35) cause a rare form of autosomal dominant PD (Gan-Or et al., 2015). VPS35 is involved in endosomal-lysosomal trafficking which is associated with autophagy. Several autosomal recessive PD genes, like parkin (*PARK2*), PINK1 (*PARK6*), DJ-1 (*PARK7*), and FBXO7 (*PARK15*) have been linked to mitophagy, the process of degradation of dysfunctional mitochondria by autophagy (Burchell et al., 2013; Gan-Or et al., 2015; Pickrell and Youle, 2015). Mutations in *LRRK2* are the most common known genetic causes for PD. LRRK2 can be degraded by macroautophagy and CMA, but the most common mutation, G2019S, is poorly degraded by this pathway (Orenstein et al., 2013). In addition, mutated LRRK2 impaired CMA leading to an accumulation of other CMA substrates, including α-SYN. Moreover, mutant LRRK2 caused an increase of autophagic vacuoles in a neuronal cellular model, proposing a more general role of LRRK2 in autophagy (Plowey et al., 2008). In a recent study, Ho et al. (2018) showed that LRRK2 mediates phosphorylation of leucyl-tRNA synthetase leading to impairment of autophagy.

After the initial finding of accumulation of autophagic vacuoles in the SN of PD patients (Anglade et al., 1997), several pathological studies suggested that macroautophagy and CMA are deregulated along with several key proteins related to macroautophagy. Increased levels of beclin-1, which is responsible for the formations and maturation of autophagosomes, and increased levels of autophagosome marker LC3II have been found in the SN of PD patients (Dehay et al., 2010; Miki et al., 2016). Likewise, decreased levels of lysosomal-associated membrane protein type 1 (LAMP1) were evident in nigral neurons in PD patients (Chu et al., 2009; Dehay et al., 2010). The impairment of CMA is associated with the pathogenesis of PD since chaperone Hsc70 and LAMP2 were less expressed in several structures of PD brains (Alvarez-Erviti et al., 2010; Murphy et al., 2015). Decreased levels of several lysosomal markers have been shown in the SN of PD patients. These include the structural protein LAMP1 (Dehay et al., 2010), the lysosomal P-type ATPase ATP13A2 (Dehay et al., 2012), GCase (Gegg et al., 2012; Murphy et al., 2014) and heat shock protein 73 (Chu et al., 2009). In addition, altered activities of lysosomal enzymes, like GCase, Cathepsin A and D have been detected in PD brains (Chu et al., 2009; Gegg et al., 2012; Murphy et al., 2014; Chiasserini et al., 2015).

Transcriptome studies have revealed deregulation of the ALP in PD brains and alterations of several autophagy-related processes, including mTOR, PI3K/AKT, and 14-3-3 protein signalings (Elstner et al., 2011; Mutez et al., 2014; Dijkstra et al., 2015). Increased levels of mTOR protein expression were found in the temporal cortex of patients with DLB, in particular in neurons displaying α-SYN accumulation (Crews et al., 2010). The alteration of other upstream autophagy-related proteins has also been demonstrated, including the immunoreactivity of UNC-51-like kinase 1 (ULK-1), ULK-2, VPS35 and autophagy/Beclin-1 regulator 1 (AMBRA1) within mature LBs, increased levels of Beclin-1, and changed subcellular localization of transcription factor EB (TFEB) (Decressac and Bjorklund, 2013; Miki et al., 2016). Moreover, in a recent study a downregulation of 6 core autophagy genes (*ULK* 3, *Atg*2A, *Atg4B, Atg5, Atg16L1*, and histone deacetylase 6), and increased protein levels of ULK1, Beclin1, and AMBRA1 were detected in peripheral blood mononuclear cells (PBMCs) of PD patients (Miki et al., 2018). These protein levels correlated with increased α-SYN levels in PBMCs. These results suggest a decrease in autophagy properties in PD patients.

##### The autophagy-lysosomal pathway and α-SYN

While α-SYN can be cleared by UPP, the main pathway for its degradation appears to be lysosomal (Webb et al., 2003; Vogiatzi et al., 2008). α-SYN can be degraded by both macroautophagy and CMA, but the structure and mutations may change the final path of degradation. Small soluble forms of α-SYN are more likely to be degraded by CMA but in the pathological condition the burden shifts to macroautophagy even though both pathways can compensate for each other.

Induction of autophagy with rapamycin leads to the clearance of overexpressed wild-type and mutant α-SYN in cell cultures (Webb et al., 2003). This study established that inhibition of macroautophagy with 3-methyladenine causes the accumulation of mutant – but not wild-type – α-SYN. In contrast, a study with PC12 cells showed an increase in both endogenous and overexpressed wild-type α-SYN when macroautophagy was inhibited with 3-methyladenine (Vogiatzi et al., 2008). Other studies with neuronal cells or transgenic mice overexpressing wild-type α-SYN showed accumulation of α-SYN only upon general lysosomal inhibition, and not in suppressed macroautophagy (Lee et al., 2004; Klucken et al., 2012). However, another study has revealed an increase of A53T α-SYN oligomers after pharmacological or molecular inhibition of macroautophagy (Yu et al., 2009). Conditional depletion of *Atg7* in DA neurons caused age-related neuronal loss, the formation of ubiquitinated protein aggregates and increase in monomeric α-SYN (Ahmed et al., 2012). In addition, α-SYN aggregates were detected in striatal axonal swellings of 20-month-old mice after depletion of *Atg7* (Friedman et al., 2012). Both studies suggested a role of macroautophagy in α-SYN turnover *in vivo*, since macroautophagy impairment caused modest alterations in endogenous α-SYN. Overall, these studies indicate that degradation of α-SYN by macroautophagy may depend on the conformation of α-SYN. It is likely that small amounts of wild-type α-SYN are degraded by CMA, but in cases of overexpression or mutations, macroautophagy becomes a more important pathway. The macroautophagic degradation of α-SYN could also depend on PTMs. Phosphorylation and SUMOylation [Small Ubiquitin-like Modifier (SUMO)] have been reported to increase α-SYN degradation by macroautophagy in yeast and PD models (Oueslati et al., 2013; Shahpasandzadeh et al., 2014; Tenreiro et al., 2014). Inside the lysosome, α-SYN is mainly degraded by Cathepsin D, and overexpression of the mutant form of this protease leads to increased levels of α-SYN (Crabtree et al., 2014). In addition, Cathepsin D knockout mice exhibited an accumulation of higher molecular weight α-SYN species (Cullen et al., 2009). Overexpression of mutant A53T α-SYN has been reported to enhance autophagic flux which causes increase in autophagic vacuoles and macroautophagic degradation (Cuervo et al., 2004; Xilouri et al., 2009; Choubey et al., 2011). Similar effects have been reported with wild-type α-SYN, although to a lesser extent. α-SYN can also inhibit macroautophagy via interaction with Rab proteins leading to Atg9 mislocalization (Winslow et al., 2010). Furthermore, α-SYN has been shown to enhance mitophagy. In a transgenic mouse model expressing A53T specifically in DA neurons, the induction of mitophagy was detected (Chen et al., 2015). In the cell culture model, α-SYN overexpression caused increased mitophagy leading to neuronal death (Choubey et al., 2011). However, elevated macroautophagic flux was evident in primary midbrain neurons overexpressing wild-type and A53T α-SYN, without significant alterations in mitophagy (Koch et al., 2015).

Besides neuronal cells, the relation of α-SYN and autophagy has also been demonstrated in other cell types. DJ-1 knockdown microglia exhibited an impaired uptake of α-SYN and had lower autophagy-dependent degradation of p62 and LC3 proteins (Nash et al., 2017). In immortalized astrocyte cell lines overexpressing wild-type, A30P and A53T mutant α-SYN showed decreased LC3-II and increased p62 protein levels, suggesting the inhibition of autophagy (Erustes et al., 2018). In addition, iPSC-derived astrocytes with *LRRK2* G2019S mutation accumulated α-SYN and had impaired macroautophagy and dysfunctional CMA (di Domenico et al., 2019). PD astrocytes displayed LAMP2A positive vesicles all around the cell body, whereas in control lines the vacuoles were in the perinuclear area. In addition, α-SYN co-localized with LAMP2A receptor in PD astrocytes. LAMP1 -positive vesicles were also found throughout the cell in PD astrocytes, and there was an increase in autophagic vacuoles. Furthermore, higher basal levels of LC3-II, p62 and impaired autophagic flux were detected from PD astrocytes.

The link between α-SYN and CMA was initially established in purified lysosomes demonstrating that α-SYN could be actively degraded by CMA (Cuervo et al., 2004). Interestingly, A30P and A53T α-SYN mutations had higher affinity to LAMP2A and blocked and totally impaired the CMA pathway. Since then, the higher affinity of the mutant α-SYN to LAMP2A was confirmed in neuronal cultures and other cell culture models (Vogiatzi et al., 2008; Alvarez-Erviti et al., 2010). In the neuronal systems, the inhibition of CMA leads to the formation of high molecular weight or detergent-insoluble oligomeric α-SYN conformations (Vogiatzi et al., 2008). Also, in mice where α-SYN expression was enhanced with paraquat or transgenic overexpression, the intralysosomal content of α-SYN was increased as well (Mak et al., 2010). The overexpression of α-SYN in mice also led to upregulation of LAMP2A and hsc70. Another study with mice with VPS35 deficiency or expression of PD-linked mutation D620N showed accumulation of α-SYN in DA neurons and DA degeneration (Tang et al., 2015). This was accomplished by an impaired endosome-to-Golgi retrieval of LAMP2A leading to decreased levels of LAMP2A and a reduced α-SYN clearance. In Drosophila, which lacks CMA, neuronal expression of human LAMP2A protected against starvation and oxidative stress and delayed the locomotor decline in aging flies (Issa et al., 2018). LAMP2A also alleviated the progressive locomotor and oxidative defects induced by neuronal expression of PD-associated human A30P α-SYN. LAMP2A stimulated autophagy in adult Drosophila, and neuronal expression of LAMP2A upregulated levels of Atg5.

PTMs can affect the degradation of α-SYN through CMA. Oxidation and nitration of α-SYN slightly inhibited the CMA, whereas phosphorylation and exposure to dopamine almost completely block the CMA degradation system. However, only dopamine-modified α-SYN blocks the degradation of other substrates (Martinez-Vicente et al., 2008). The same study reported that CMA could degrade only monomeric or dimeric α-SYN, but not oligomers. Blocking the CMA by aberrant forms of α-SYN can also have a toxic effect and impact other degradation pathways. PD-linked mutations like A30P and A53T or dopamine-modified wild-type α-SYN can inhibit the function of CMA leading to activation of macroautophagy and increased toxicity in cells (Martinez-Vicente et al., 2008; Xilouri et al., 2009).

Recently, micro-RNAs (miRNAs) have been implicated in CMA function and α-SYN clearance. Several miRNAs have been described to target LAMP2A and Hsc70 and decrease α-SYN degradation (Alvarez-Erviti et al., 2013; Li et al., 2014). The initial study found four miRNAs that reduce LAMP2A levels, and three that decreased Hsc70 levels. This was accompanied by increased accumulation of α-SYN in SH-SY5Y neuroblastoma cells. These miRNAs were also found up-regulated in brains of PD patients and correlated with decreased protein levels of CMA (Alvarez-Erviti et al., 2013).

##### Autophagy enhancing agents as a potential therapeutic strategy for PD

Because ALP is an important pathway in α-SYN degradation, the opportunity to use autophagy enhancement as a strategy against α-SYN aggregation in PD has raised considerable interest. Pioneering studies with rapamycin and other macroautophagy enhancing agents have demonstrated an increased α-SYN clearance in several PD models. However, the selectivity of these early autophagy enhancers is limited. Selective targeting of ALP components, like TFEB, lysosomes, and CMA, may provide more potential for development of new therapies for PD. The main findings are listed in Table 1, and for more details see the literature (Moors et al., 2017).

**Table 1:** Commonly used autophagy enhancing agents.

	Target	Agent	PD model	Effect	References
Autophagy	mTORC1	Rapamycin and	α-SYN overexpressed SH-SY5Y	↓Phospho-Ser129 α-SYN levels	Pérez-Revuelta et al., 2014
		analogs	Rotenone-exposed SH-SY5Y cells	↓Cell death, ↓Mitochondrial dysfunction	Pan et al., 2009; Xiong et al., 2011; Hou et al., 2015
			6-OHDA and MPTP treated PC12	↓Cell death	Malagelada et al., 2010
			MPTP mice	↓Cell death	Dehay et al., 2010; Malagelada et al., 2010
			αα -SYN transgenic mice	↓Cell death	Crews et al., 2010
			α -SYN transgenic rats	↓Cell death	Decressac et al., 2013
			A53T α-SYN transgenic mice	↓Cell death	Bai et al., 2015
			6-OHDA mice	↓Levodopa-induced dyskinesia	Santini et al., 2009
			6-OHDA rats	↓Levodopa-induced dyskinesia	Decressac and Bjorklund, 2013
	AMPK	Metformin	MPTP mice	↓Cell death, α-SYN levels	Katila et al., 2017
				↑Neurotrophic factors	
			α-SYN overexpressed SH-SY5Y	↓Phospho-Ser129 α-SYN levels	Pérez-Revuelta et al., 2014
	Beclin-1	PREP inhibitor	A30P α-SYN transgenic mice	↓Oligomeric α-SYN, ↑Striatal DA levels	Savolainen et al., 2014
		Isorhynchophylline	N2a cells transfected with WT, A53T and A30P α-SYN	↑α-SYN clearance	Lu et al., 2012
			Embryonic DA neurons	↑α-SYN clearance	Lu et al., 2012
			WT, A30P, and A53T α-SYN expressing PC12 cells	↑α-SYN clearance, ↓α-SYN accumulation	Webb et al., 2003
			α-SYN transgenic mice	↑α-SYN clearance, ↓α-SYN accumulation	Miki et al., 2016
	TFEB	2-HPβCD	Human neuroglioma cells transfected with α-SYN	↑α-SYN clearance	Kilpatrick et al., 2015
	SLC2A	Trehalose	Rotenone-treated rats	↓Cell loss	
			Rotenone-treated PC12 cells	↓Cell loss, ↑α-SYN clearance	Wu et al., 2015
			M MPTP mice	↓Cell loss, ↓Neuroinflammation,	Sarkar et al., 2014
				↓Motor deficits	
			A53T α-SYN overexpression in rats	↓Cell loss, ↓Motor deficits, ↑α-SYN clearance	He et al., 2016
			WT and A53T α-SYN expressed PC12 cells	↑α-SYN clearance	Sarkar et al., 2007; Lan et al., 2012
			NB69 human neuroblastoma cells	↑α-SYN clearance	Casarejos et al., 2011
			A53T α-SYN overexpressing mice	↑Detergent-insoluble α-SYN clearance	Tanji et al., 2015
Lysosomes	GCase	Ambroxol	GBA1 mutant fibroblasts	↑Lysosomal function, ↑GCase activity ↓Oxidative stress	McNeill et al., 2014; Ambrosi et al., 2015
			Primary cortical neurons	↑GCase activity, ↑TFEB	Magalhaes et al., 2018
			α α-SYN transgenic mice	↑GCase activity, ↓α-SYN levels	Migdalska-Richards et al., 2016


*mTORC1, mammalian target of rapamycin complex 1 or mechanistic target of rapamycin complex 1; PREP, prolyl oligopeptidase; 2-HPβCD, 2-hydroxypropyl-β-cyclodextrin; α-SYN, alpha-synuclein; DA, dopaminergic; ROS, reactive oxidative stress; WT, wild-type; 6-OHDA, 6-hydroxydopamine; MPTP, 1-methyl-4-fenyl-1,2,3,6-tetrahydropyridine; Gcase, glucocerebrosidase; TFEB, transcription factor EB; SLC2A, (GLUT) family of glucose.*

The most studied and used macroautophagy-enhancer is rapamycin which inhibits mTORC1 signaling (Bové et al., 2011). Rapamycin has been shown to reduce α-SYN accumulation in wild-type, A30P, or A53T α-SYN expressing PC12 cells and in mice (Crews et al., 2010) and rats (Decressac and Bjorklund, 2013) with overexpressed α-SYN. Rapamycin also improved the motor function in mice with overexpressed A53T α-SYN (Bai et al., 2015). The drawback of mTORC1 inhibition is the interference with numerous other pathways. Prolonged treatment with rapamycin can inhibit mTORC2 and stimulate other cellular pathways, including cell survival mechanisms (Bové et al., 2011). The activation of macroautophagy can be achieved by activating AMPK, leading to downstream inhibition of mTORC1. Several agents which act through this pathway have been described, such as metformin, 5-aminoimidazole-4-carboxamide ribonucleotide (AICAR) and resveratrol (Curry et al., 2018). Metformin, commonly used to treat *Diabetes Mellitus*, showed neuroprotective effects in *in vitro* and *in vivo* models of PD (Ng et al., 2013; Dulovic et al., 2014; Patil et al., 2014). Metformin also decreased phosphorylated levels of α-SYN in SH-SY5Y cells and MPTP-treated mice (Pérez-Revuelta et al., 2014; Katila et al., 2017). Another agent that affects the AMPK signaling is trehalose, which inhibits members of the SLC2A (*GLUT*) family of glucose transporters leading to AMPK-dependent increase of macroautophagy (DeBosch et al., 2016). Trehalose-induced autophagy has shown to increase cell survival and α-SYN clearance in cell lines and in multiple *in vivo* models (Sarkar et al., 2007, 2014; Rodríguez-Navarro et al., 2010; Lan et al., 2012; Tanji et al., 2015; He et al., 2016). However, a recent study did not find improvement in neuronal survival after exposure to α-SYN pre-formed fibrils (Redmann et al., 2017). Although increasing autophagy by AMPK pathway has shown beneficial effects, AMPK is involved in several other cellular functions, and its modulation is likely to induce unwanted effects.

Recently, several other agents acting through an mTOR-dependent pathway have been studied in cell cultures and PD animal models. Sheng et al. (2017) showed that uric acid treatment increased autophagy in PC12 cell in dose- and time-dependent manners. Moreover, uric acid reduced α-SYN accumulation in PC12 cells overexpressing wild-type or A53T mutant α-SYN. *In vivo*, uric acid modulated autophagy markers increased the autophagosome/autolysosome formation and reduced α-SYN accumulation in the midbrain of SNCA A53T transgenic mice. Suresh et al. (2017) showed that a novel autophagy modulator 6-Bio alleviated α-SYN toxicity. In yeast and mammalian cell lines, 6-Bio induced autophagy and enhanced autolysosome formation which resulted in α-SYN degradation and clearance. *In vivo* studies with a MPTP mouse model demonstrated that 6-Bio has a neuroprotective activity, enhances autophagy and clearance of toxic protein aggregates and ameliorates MPTP-induced behavioral deficits. The results demonstrated that 6-Bio modulates autophagy in a GSK3B-dependent manner and the induction of autophagy in mammalian cells appears to be mTOR dependent.

Instead of general activation of macroautophagy, targeting selective ALP components including Beclin-1, TFEB, and lysosomes have been tried. Activation of Beclin-1 induces autophagosome formation and initiation of autophagy. Overexpression of Beclin-1 has been shown to reduce accumulation of α-SYN in PC12 cells and mice with overexpressed α-SYN (Spencer et al., 2009; Wang et al., 2016). In addition, the drug-induced activation of Beclin-1 has been demonstrated to increase autophagy and promotes α-SYN clearance in neuronal cell lines and PD animal models (Lu et al., 2012; Savolainen et al., 2014). One attractive target to stimulate macroautophagy downstream of mTORC1 is modulating transcriptional levels of TFEB. TFEB regulates macroautophagy and lysosomes and acts as a link between upstream signaling pathways (Settembre et al., 2011). Overexpression of TFEB eliminated α-SYN oligomers and rescued midbrain DA neurons from α-SYN toxicity in overexpressing rats (Decressac and Bjorklund, 2013). Another strategy for stimulating ALP in PD is a direct modulation of lysosomes. The potential of targeting the lysosome system has been demonstrated with acidic nanoparticles which were able to stimulate lysosomal degradation and revert the lysosomal dysfunction in genetic PD models (Baltazar et al., 2012; Bourdenx et al., 2016). Ambroxol, AT2101 (isofagomine) and histone deacetylase inhibitors can correct the folding of GCase and therefore increase the GCase and lysosome function (Blanz and Saftig, 2016). The small-molecule chaperones have been demonstrated to enhance GCase activity, improve lysosomal function and enhance α-SYN clearance in preclinical models of PD (Steet et al., 2006; Khanna et al., 2010; Sun et al., 2012; Yang et al., 2013; McNeill et al., 2014; Richter et al., 2014; Ambrosi et al., 2015). Especially ambroxol is widely studied presently. In mice overexpressing α-SYN or heterozygous L444P mutation in *CBA1*, ambroxol treatment increased the GCase activity while decreasing phosphorylated and endogenous levels of α-SYN (Migdalska-Richards et al., 2016, 2017b). In non-human primates, ambroxol increased brain GCase activity (Migdalska-Richards et al., 2017a). In patients with Gaucher disease, ambroxol was able to cross the blood–brain barrier and high-dose oral administration was safe and well-tolerated (Narita et al., 2016).

Currently, ambroxol is in phase II clinical trials tested for treatment of PD and PD with dementia (ClinicalTrials.gov Identifier: NCT02941822 and NCT02914366, respectively) (Silveira et al., 2019).

Downstream targeting of the CMA components presents an alternative approach to develop new strategies for PD. Induced overexpression of LAMP2A in human SH-SY5Y cells, rat primary cortical neurons *in vitro* and nigral DA neurons *in vivo* decreased α-SYN accumulation and protected α-SYN-induced DA degeneration (Xilouri et al., 2013). Retinoic acid alpha receptors have been identified as CMA inhibitors, and synthetic derivatives of all-*trans*-retinoic acid were developed to reverse this effect (Anguiano et al., 2013). These derivates specifically stimulated CMA and LAMP2A was identified as one of the targets.

## Role of ER Stress in PD as a Result of Dysfunctional Cellular Proteostasis

Endoplasmic reticulum is the first component of the secretory pathway mainly responsible for protein synthesis, post-translational processing and folding of newly synthesized proteins. The proteins are then transported to their final destinations in membrane-bound vesicles. Disturbance in any of these functions including proper folding capacity and disposal of misfolded proteins leads to ER stress and activation of intracellular signal transduction pathway that is essentially intended to re-establish ER homeostasis. These biological processes are collectively called the UPR. Inability to restore ER functions induces cell death via apoptosis. Growing evidence from studies in human PD post-mortem brain, additionally to genetic and neurotoxin models, suggests that ER stress is a common feature in PD that contributes to PD pathology. Recently, the generation of neuronal cultures from iPSCs derived from PD patients indicated that ER stress leads to the accumulation of ER-associated degradation (ERAD) substrates and placed this ER dysfunction as an early component of PD pathogenesis (Chung et al., 2013; Heman-Ackah et al., 2017).

### Causes of ER Stress in PD

The ER is crucial for protein folding, trafficking to the Golgi, UPR, and calcium buffering. The imbalance between the load on ER functions and ER capacity leads to ER stress. In PD, the mechanisms leading to ER stress and the actual role of the UPR in degeneration of the DA neurons are the object of intensive research. Oligomeric α-SYN has been shown to accumulate in the ER in animal models and PD patient brains (Colla et al., 2012). The aggregation of α-SYN induces ER stress, which eventually results in inflammation and neurodegeneration.

A number of studies have shown that α-SYN affects Rab1, a protein involved in trafficking components from the ER to the Golgi. Over-expression of Rab1 in animal models of PD reduced stress levels and protected DA neurons against degeneration (Coune et al., 2012). Further, α-SYN directly interacts with nascent activating transcription factor 6 (ATF6), effectively preventing its association with COPII vesicles that generally transfer proteins to the Golgi. This has specific implications: (1) interfering with the Rab1 protein could lead to accumulation of unfolded proteins in the ER, and (2) inhibition of ATF6 would generally stop the ERAD, triggering the cell to signal apoptosis (Credle et al., 2015). Other studies are linking PD genes with alteration of the secretory pathway, including LRRK2, Parkin, DJ-1, ATP13A2 (Mercado et al., 2013), and VPS35 (Zimprich et al., 2011), which may result in pathological levels of ER stress contributing to the etiology of the disease. Furthermore, increase in cytoplasmic Ca^2+^ levels induced by 6-hydroxydopamine (6-OHDA) (a toxin capable to generate, *in vitro* and *in vivo*, some features of PD-associated neurodegeneration) was detected in 6-OHDA-treated rats. Pretreatment with ryanodine or ER stress inhibitor 4-PBA inhibited RyR receptor Ca^2+^ channels and protective midbrain DA neurons from degeneration (Huang et al., 2017).

### UPR Response in PD

**FIGURE 2:**
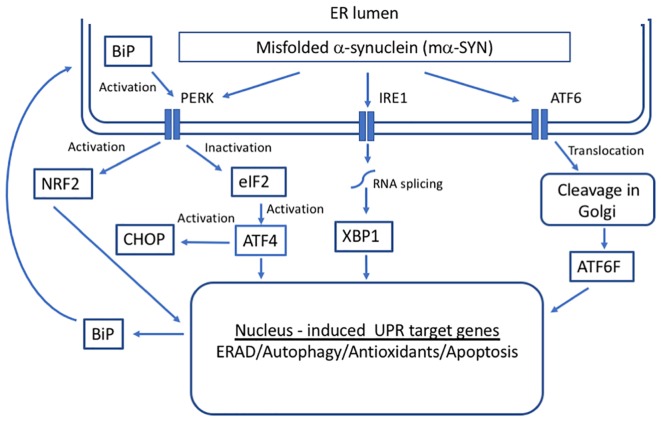
Unfolded protein response (UPR) in response to alpha-synuclein misfolding. Three transmembrane proteins have been identified as sensors of unfolded proteins in the ER in mammalian cells: IRE1 (inositol-requiring protein 1), ATF6 (activating transcription factor 6), and PERK (protein kinase RNA-like ER kinase). Upon the accumulation of mα-SYN, BiP dissociates from UPR sensors inducing their activation that leads to the transcription of genes whose protein products increase the folding capacity of the cell.

The primary function of the UPR is a maintenance of ER protein homeostasis. When cells undergo constant ER stress, the UPR is responsible for the elimination of damaged cells through apoptotic mechanisms, some of which appear to be specific to ER stress and others that are included in general apoptotic pathways (Xu et al., 2005). The activation of UPR depends on three ER stress sensors proteins, protein kinase RNA-like endoplasmic reticulum kinase (PERK) receptor, inositol-requiring enzyme 1 (IRE1), and ATF6 (Schröder and Kaufman, 2005) (Figure 2). Under normal physiological conditions, all three effectors are negatively regulated by the ER chaperone glucose-regulated protein 78/ binding immunoglobulin protein (GRP78/BiP), which suppresses their activity by binding to their luminal ends (Bertolotti et al., 2000).

Under conditions of ER stress and increase in unfolded proteins, BiP dissociates from UPR sensors inducing their activation. Activation of the ER pathways helps to fight the cellular stress through the combined actions of suppressing the translation of new proteins, inducing ER chaperones that promote protein refolding and activating the proteasome to degrade misfolded/unfolded proteins. There is direct evidence that GRP78/BiP levels are increased in cell as well as animal models of PD forming a complex with α-SYN (Bellucci et al., 2011). Moreover, it has been demonstrated that aging leads to a significant decline in GRP78/BiP expression (up to 40%; Naidoo, 2009). However, under chronic ER stress, UPR sensors shift their signaling toward induction of cell death by apoptosis (Urra et al., 2013).

#### PERK Signaling

PERK is a type I ER transmembrane protein kinase with a luminal domain and a cytoplasmic domain that has kinase activity (Liu et al., 2002). Upon ER stress, BiP releases the luminal domain of PERK, which then dimerizes and autophosphorylates to become active (Harding et al., 1999). Following trans-autophosphorylation, this kinase phosphorylates the alpha subunit of eukaryotic initiation factor-2 (eIF2), inactivating it by Ser-51 phosphorylation and attenuating protein translation. This inhibitory effect of translation helps to alleviate ER stress by decreasing the overload of misfolded proteins and thereby protecting the cells under conditions where proteins cannot achieve proper folding (Fels and Koumenis, 2006). This event leads to activating transcription factor 4 (ATF4). The UPR-related transcriptional factor ATF4 upregulates a subset of genes that control oxidative stress, metabolism, protein folding, and glutathione biosynthesis (Harding et al., 2000). Important targets of ATF4 include *NRF2*, which regulates the functions of a variety of antioxidant genes (He et al., 2001), and *CHOP*, which conversely is a key in the activation of apoptotic pathways and cell death (Han et al., 2013). In PD patients, the activation of PERK/ATF4 signaling is observed in different brain areas. α-SYN has been shown to accumulate within the ER of nigral DA cells, directly activating the PERK/eIF2α signaling and increasing the expression of ATF4 (Bellucci et al., 2011). The activation of this pathway overlapped with pro-apoptotic changes.

Further evidence supporting this pathway comes from PD-associated gene studies. By inducing the A53T α-SYN mutation to PC12 cells, UPR activates CHOP and GRP78/BiP by increasing their expression and increases the phosphorylation of eIF2α (Smith et al., 2005). Interventions to block ER stress and caspase activity using inhibitors, and to knock down the expression of caspase-12 using siRNA, protected against A53T α-SYN -induced cell death (Smith et al., 2005). In PINK1- and Parkin-associated PD models, mitofusins cause enhanced ER stress signaling, by interconnecting damaged mitochondria to the ER membranes (Celardo et al., 2016). PERK signaling inhibition, either pharmacological or genetic suppression, was beneficial in these experimental models of PD (Celardo et al., 2016). LRRK2 mutations also cause familial PD by accumulation and aggregation of α-SYN and ubiquitinated proteins over time mainly due to the impairment of protein degradation pathways (Tong et al., 2010). This is likely to result in the build-up of unfolded proteins leading to ER stress, although there is little evidence of UPR activation yet. On the other hand, studies with *GBA1* gene mutations revealed that treatment with chemical chaperones (ambroxol and isofagomine) can combat *GBA*-mediated ER stress by increasing *GBA* levels and activity in fly models and in fibroblasts from PD patients (Sanchez-Martinez et al., 2016). In rodent models of PD, *ATF4* upregulation in DA neurons of SN resulted in severe nigrostriatal degeneration caused by activating caspase 3/7-dependent pathway (Gully et al., 2016). On the other hand, Grp78/BiP over-expression exerted neuroprotective effects in a rat model of PD (Gorbatyuk et al., 2012).

#### IRE1 Signaling

IRE1 is a type I ER transmembrane sensor and cell fate executor. IRE1 gets activated in response to the accumulation of misfolded proteins by autophosphorylation. The activation induces RNase activity that is consequently needed for unconventional splicing of X-box binding protein 1 (XBP1) (Calfon et al., 2002). Spliced XBP1 translocates to the nucleus where it commands transcription of genes responsible for quality control, protein folding, lipid synthesis and ERAD pathway (Sriburi et al., 2004). In PD, XBP1 controls the survival of DA neurons (Valdés et al., 2014). The developmental ablation of XBP1 preconditions DA neurons against the effect of the neurotoxin, 6-OHDA (Mollereau et al., 2014, 2016). The effect is specific to SNpc, as it is not seen in other brain regions. Contrary, reduction of XBP1 in DA neurons of adult mice caused ER stress with CHOP induction leading to degeneration (Valdés et al., 2014), highlighting the critical role of XBP1 depending on the development stage. In addition, a gene therapy approach using adeno-associated viral vectors to deliver XBP1 active form to the SNpc confers protection of DA neurons against 6-OHDA-mediated toxicity (Valdés et al., 2014). Moreover, XBP1 transgene delivered to mouse striatum using recombinant adenoviral vectors protected DA neurons against MPTP-induced degeneration (Sado et al., 2009). XBP1 is also protective when it is delivered in neural stem cells transfected with this transcription factor resulting in increased survival and improved behavior in a rotenone-induced rat model of PD (Si et al., 2012). Among other functions, XBP1 and ATF6 mediate the transcription of BiP. While the overexpression of this chaperone protects DA neurons and increases motor performance in a rat model of PD, the age-related decline in BiP expression as well as siRNA-mediated downregulation, increases DA neuron vulnerability to α-SYN in the same PD model (Salganik et al., 2015).

#### ATF6 Signaling

ATF6 is a type II ER transmembrane protein. Upon the accumulation of misfolded proteins in the ER, ATF6 moves to the Golgi apparatus where it is cleaved by two proteases (Haze et al., 1999). The cytosolic domain of ATF6 is translocated to the nucleus where it activates the transcription of ER chaperones (Gotoh et al., 2002). Contrarily, reduced levels of ATF6 in the nucleus of cells due to the impairment in ATF6 trafficking to the Golgi are likely to trigger apoptosis (Credle et al., 2015). In PD, α-SYN directly targets ATF6 and inhibits ATF6 processing leading to an impaired up-regulation of ERAD genes, which sensitizes cells to apoptosis (Credle et al., 2015). In ATF6α knockout animals, the accumulation of ubiquitin-positive inclusions and enhanced loss of DA neurons induced by MPTP, a PD-triggering neurotoxin, was detected (Egawa et al., 2011). This suggests that activation of the UPR has an important adaptive function to maintain protein homeostasis in this model. ATF6 mainly controls the levels of BiP and ERAD elements rather than development and survival of DA neurons in mice under resting conditions (Egawa et al., 2011).

## Suppression of Excessive Protein Oxidative Folding as an Alternative Solution for Lowering ER Stress

As indicated above, ER stress is increasingly implicated in PD, and emerging evidence highlights the complexity of the UPR, with both protective and detrimental components being described. Mild insults increase the activity of chaperones, such as protein disulfide isomerase (PDI) that is responsible for the oxidative folding through formation of disulfide bonds in proteins (Rao and Bredesen, 2004). To promote correct disulfide bond formation in unfolded/misfolded proteins, the redox environment in the ER is oxidatively maintained (Hwang et al., 1992). In neurons, the increased activity of PDI represents an adaptive response that is induced to protect the cells (Haynes et al., 2004). In contrast, recent studies have revealed alternative roles for PDI in neurodegenerative diseases (Hoffstrom et al., 2010; Lehtonen et al., 2016).

**FIGURE 3:**
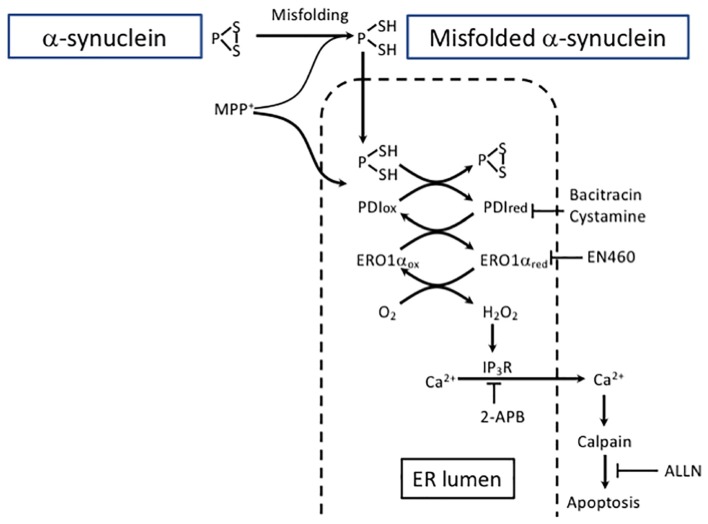
Excessive protein refolding in ER leads to oxidative stress and apoptosis. Depending on the structure, aggregates can be degraded either by macroautophagy or CMA. Alternatively, misfolded α-SYN undergoes refolding in the ER. However, excessive refolding upregulates PDI reduction. Re-oxidation of PDI is linked with an increase in hydrogen peroxide generation causing dysregulation of IP3R permeability and an increase in cytosolic calcium. Calcium release from the ER may activate calpain and eventually lead to apoptosis. The pharmacological inhibition of PDI by bacitracin or cystamine prevents ER redox imbalance and downstream proapoptotic events. The inhibition of the ERO1 catalyzed re-oxidation of PDI by EN460 results in a protective effect similar to PDI inhibitor.

In PD, ER homeostasis is disrupted in DA neurons in SNpc and PDI co-localizes with α-SYN in LBs (Conn et al., 2004). We have recently demonstrated that treatment with 1-methyl-4-phenylpyridinium (MPP+), a neurotoxin associated with PD, upregulated the expression of α-SYN and PDI in human neuroblastoma SH-SY5Y cells and that α-SYN co-localized with PDI. The α-SYN accumulation not only activated PDI but resulted in the accumulation of a reduced form of PDI due to an increasingly reduced ER redox environment (Figure 3).

Protein disulfide isomerase inhibitors bacitracin and cystamine prevented the accumulation of α-SYN and MPP+-induced reductive shift in the ER by hindering PDI excessive reduction (Lehtonen et al., 2016). Moreover, the data suggest that redox misbalance and hydrogen peroxide production due to PDI re-oxidation in the ER are the outcome of a severe toxic insult caused by α-SYN accumulation. Calpain is a Ca^2+^ -sensitive non-lysosomal protease reported to be disruptive in SN of PD patients as well as in experimental PD models (Crocker et al., 2003). In the model described by Lehtonen et al. (2016), the release of Ca^2+^ was succesfully blocked not only by 2-aminoethoxydiphenyl borate (2-APB), an inositiol-3-phosphate receptor (IP3R) inhibitor, but also by bacitracin, a PDI inhibitor, and it promoted neuroblastoma cell survival. Additionally, ALLN (*N*-acetyl-leu-leu-norleual, *N*-acetyl-L-leucyl-L-leucyl-L-norleucinal), a calpain I inhibitor, protected these cells from MPP+ toxicity. Overall, these results are in line with a previously published study using a PC12 cell model of Huntington’s disease showing the potential of PDI inhibitors to suppress the cells’ death induced by misfolded proteins (Hoffstrom et al., 2010). Importantly, a beneficial effect of PDI inhibition is coupled with consecutive enhancement of autophagy that is turned on to support cell survival. Furthermore, PDI inhibition also protected against MPP+ -induced DA neurodegeneration in *Caenorhabditis elegans*.

Collectivelly, excessive protein refolding taking place in the ER leads to an increase in the reduced form of PDI and to the activation of the PDI-Ero1 cycle, causing overproduction of hydrogen peroxide and promoting generation of ROS. These events lead to the dysregulation of IP3R that causes an increase in cytosolic calcium followed by calpain activation-induced apoptosis. In contrast, PDI inhibition prevents ER redox imbalance and enhances the autophagic clearance pathway. Therefore, when considering therapeutic approaches, it is necessary to take into account the balance between ER-linked refolding or/and alternative protein clearance by autophagy.

## Author Contributions

All authors listed have made a substantial, direct and intellectual contribution to the work, and approved it for publication.

## Conflict of Interest Statement

The authors declare that the research was conducted in the absence of any commercial or financial relationships that could be construed as a potential conflict of interest.
